# Impact of butorphanol versus sufentanil on postoperative cognition and inflammation in elderly: a pilot study

**DOI:** 10.3389/fnagi.2024.1395725

**Published:** 2024-06-07

**Authors:** Qiannan Wen, Defeng Sun, Lin Yang, Yuexian Li

**Affiliations:** ^1^Department of Anesthesiology, The First Affiliated Hospital of Dalian Medical University, Dalian, China; ^2^Department of Neuroelectrophysiology, The First Affiliated Hospital of Dalian Medical University, Dalian, China

**Keywords:** butorphanol, inflammation, post-operative cognitive dysfunction, randomized controlled trial, sufentanil

## Abstract

**Background:**

This randomized controlled trial aimed to compare the effects of butorphanol and sufentanil on early post-operative cognitive dysfunction (POCD) and systemic inflammation in older surgical patients.

**Methods:**

Patients (aged 65 years or above) undergoing surgeries with general anesthesia were randomized to either the butorphanol group (40 μg/kg during anesthesia induction) or the sufentanil group (0.4 μg/kg). Cognitive function changes during the perioperative period were assessed using the Mini-Mental State Examination (MMSE) and the Montreal Cognitive Assessment (MoCA) scale up to 3 days after surgery. POCD was defined as a *Z*-score or composite *Z*-score greater than 1.96 for both MMSE and MoCA scores. Circulating inflammatory factors, including tumor necrosis factor-alpha (TNF-α), interleukin 1 beta (IL-1β), and interleukin 10 (IL-10), were measured using enzyme-linked immunosorbent assay.

**Results:**

The study included 114 patients (median age: 71 years, 57.7% male). Compared to sufentanil, butorphanol significantly reduced the incidence of POCD on the first (11.5% versus 32.7%, *p* = 0.017) and third day (3.8% versus 15.4%, *p* = 0.046) after surgery. Additionally, patients receiving butorphanol had significantly lower circulating levels of TNF-α and IL-1β at the time of discharge from the post-anesthesia care unit and on the first and third day after surgery (*p* < 0.05 for all comparisons). Furthermore, circulating IL-10 levels were significantly higher in patients receiving butorphanol (*p* < 0.05 for all comparisons).

**Conclusion:**

Administration of butorphanol during anesthesia induction, as opposed to sufentanil, was associated with a significant reduction in the early incidence of POCD in older surgical patients, possibly attributed to its impact on systemic inflammation.

**Clinical trial registration:** The present study was registered in the China Clinical Trial Center (ChiCTR2300070805, 24/04/2023).

## Introduction

Post-operative cognitive dysfunction (POCD) is one of the most common perioperative complications in older surgical patients, which may occur in 20–50% of post-surgery patients (Kotekar et al., 2018; Lin et al., 2020). The incidence of POCD has been related to a few adverse outcomes, such as prolonged hospitalization (Li et al., 2022) and possibly increased risk of long-term mortality (Dalefield et al., 2022). Therefore, it is clinically significant to determine the mechanisms of POCD and develop effective prophylactic measures (Kotekar et al., 2018). The pathogenesis of POCD is multifactorial, which involves patient characteristics (age, educational level, mental health, and comorbidities), duration and complexity of surgery, and anesthesia-related factors (Kotekar et al., 2018). Accumulating evidence has suggested multiple pathophysiological mechanisms underlying POCD, including neuroinflammation, oxidative stress, autophagy disorder, impaired synaptic function, and lack of neurotrophic support (Lin et al., 2020). Among them, inflammation plays a vital role in the pathogenesis of POCD (Li et al., 2022). On the one hand, local surgical trauma stimulates the production and release of inflammatory cytokines such as tumor necrosis factor-alpha (TNF-α), interleukin 1 (IL-1), and interleukin 6 (IL-6) (Qian et al., 2005; Zhu et al., 2014). On the other hand, systematic inflammation increases the permeability of the blood–brain barrier, leading to changes in neuronal activity and synaptic transmission in the central nervous system, which finally leads to the pathogenesis of POCD (Qian et al., 2005; Zhu et al., 2014). These findings may highlight the potential importance of systematic inflammation as a target to prevent the incidence of POCD.

Kappa-opioid receptor (KOR) is conventionally recognized by its effect on analgesia (Luan et al., 2021; Huang et al., 2022). Subsequent studies showed that KOR also confers other physiological functions, such as anti-nociception, cardiovascular, anti-pruritic, diuretic, antitussive, and anti-inflammatory effects (Dalefield et al., 2022). A recent preclinical study showed that KOR antagonist potentiates levels of pro-inflammatory cytokines (IL-1β, IL-6) in blood following administration of lipopolysaccharide in mice, suggesting the potential exaggerating effect of KOR antagonist on systemic inflammation (Wu et al., 2022). In addition, a study in the rat model of cardiopulmonary bypass surgery suggested that KOR agonists improved POCD in rats with CPB, accompanied by reduced blood levels of inflammatory factors such as TNF-α, IL-1β, and IL-6 (Parikh et al., 2014). These findings suggest that KOR agonists may effectively prevent POCD by attenuating systematic inflammation.

Butorphanol is an antagonist of the opioid receptor, which acts mainly on activating KOR (Commiskey et al., 2005). Accumulating evidence suggests that butorphanol has multiple advantages over other medications for perioperative analgesias, such as little effect on respiration and circulation and minimal influence on gastrointestinal reaction and immune suppression (Zhu and Zhang, 2021). However, limited clinical studies have been conducted to explore the impact of butorphanol on POCD. Therefore, in this study, we hypothesized that butorphanol, acting as an antagonist of KOR, might exhibit greater effectiveness than sufentanil in preventing POCD in older surgical patients under general anesthesia. To rigorously test this hypothesis, a head-to-head randomized controlled trial (RCT) was conducted to investigate the potential benefits of butorphanol on POCD and explore whether these benefits are associated with its inhibitory effect on systemic inflammation.

## Methods

The study protocol was approved by the Ethics Committee of the affiliated Hospital of Dalian Medical University (Approval number: PJ-KY-2022-314) and registered at the Chinese Clinical Trial Registration Center (registration code: ChiCTR2300070805,^1^ Principal investigator: Qiannan Wen, Date of registration: 24/04/2023) before the enrollment of the patients. The study was followed the CONSORT reporting guidelines for clinical trials. All patients signed written consent before the study procedure was initiated. The patients were fully informed about the purpose of the trial, per the Declaration of Helsinki.

### Patient inclusion and exclusion criteria

Patients aged 65 years or older who underwent surgeries with general anesthesia between May 2022 and December 2022 were included in this randomized controlled trial (RCT). The following inclusion criteria were applied: (1) American Society of Anesthesiologists (ASA) Class I to III, (2) estimated surgery time of ≥2 h, (3) body mass index (BMI) ≤ 30 kg/m^2^ (Feinkohl et al., 2016), (4) preoperative Mini-Mental State Examination (MMSE) score > 23 and Montreal Cognitive Assessment (MoCA) score ≥ 26, (5) no contraindications for the use of butorphanol, and (6) absence of severe neurologic or psychiatric diseases, severe abnormalities in liver or renal functions, serious visual and auditory disorders, and no recent history of sedative, analgesic, or antidepressant use. According to general indications of the surgeries, patients with evidence of infection were not scheduled for surgeries until the infection was well controlled.

Patients with the following clinical conditions were excluded from the study: (1) perioperative red blood cell infusion of >3 units (Zhu et al., 2014), (2) post-operative infection, (3) insufficient post-operative analgesia, (4) inability to obtain a blood sample during the perioperative periods, (5) patients who were unable to complete MoCA and MMSE assessments after surgery, or (6) patients who refused to participate in this study.

### Randomization and intervention

Patients who fulfilled the above inclusion criteria were randomized to an intervention group of butorphanol (40 μg/kg during anesthesia induction) and a control group of sufentanil (0.4 μg/kg). The randomization was achieved according to the computer-generated random sequence with a ratio of 1:1.

### Protocol of anesthesia

After entering the operating room, the venous pathway of the patients was accessed. The patients were conventionally monitored for parameters including pulse of blood oxygen saturation, electrocardiogram, non-invasive blood pressure, bispectral index (BIS), and body temperature (T). Local anesthesia was administered through the most prominent point of radial artery pulsation, followed by ultrasound-guided radial artery puncture and catheterization to enable real-time invasive arterial blood pressure monitoring.

All patients underwent intravenous induction with propofol 1–3 mg/kg and cisatracurium besilate 0.2 mg/kg. According to the randomization, butorphanol 40 μg/kg or sufentanil 0.4 μg/kg was separately administered. Tracheal intubation was conducted following the loss of consciousness and muscle relaxation in the patient. Once the successful intubation was verified, the respiratory parameters were adjusted accordingly: tidal volume of 6–8 mL/kg, respiratory frequency of 12–14 times per minute, the inhalation oxygen concentration of 50%, maintenance of end-expiratory partial pressure of carbon dioxide between 35 and 40 mmHg, and then mechanical ventilation was initiated.

A combination of intravenous and inhalation methods was used for anesthesia maintaining by continuous intravenous infusion of propofol 1–3 mg/kg/h and cisatracurium besilate 0.1–0.12 mg/kg/h and inhalation of 1–2% sevoflurane. During the surgery, butorphanol or sufentanil were supplemented appropriately according to the status of the patients. The fluctuation range of mean arterial pressure (MAP) was maintained within ±20% of the baseline level, and the heart rate (HR) was maintained between 50 and 80 bpm. An inflatable heating blanket was used to provide heating and keep the core temperature of the patients >36.0°C. The BIS values were maintained between 40 and 60.

The infusion of cisatracurium besilate was discontinued approximately 45 min before the end of the surgery, and the infusion of propofol was discontinued when the surgery was finished. Subsequently, the post-operative intravenous patient-controlled intravenous analgesia (PCIA) pump was connected, and the regimen was set according to the group of the patients (butorphanol 0.15 mg/kg or sufentanil 1.5 μg/kg with normal saline to 100 mL). The parameters settings of the PCIA pump were 2 mL load, 1.5 mL/h sustained dose, 2 mL self-control analgesic doses per session, and a locking time of 15 min. Subsequently, the patients were transferred to the post-anesthesia care unit (PACU) for post-operative monitoring and management, and extubation of the tracheal catheter was performed if the indication of extubation was fulfilled. Peripheral blood samples were obtained from the patients, and the patients were sent back to the ward if the total modified Aldrete score was ≥12 and each component score was ≥1.

### Post-operative management and outcomes evaluation

Patients were followed by the same anesthesiologist after the surgery, and the same psychiatrist evaluated the post-operative cognitive function of patients. The primary outcomes of the study included the early incidence of POCD and the changes of circuiting levels of systemic inflammatory cytokines.

Briefly, the same psychiatrist used the MMSE and MoCA scales to assess the cognitive function of patients on the day before surgery (D0), the first day after surgery (D1), and the third day after surgery (D3). The total scores of MMSE and MoCA were both 30. The incidence of POCD was defined as the *Z*-value or composite *Z*-value of both the MMSE and MoCA scores of the patients >1.96 (Evered and Silbert, 2018).

Venous blood samples were obtained from each patient on the non-infusion side of the arm before surgery (C0), at the time of discharge from the PACU (C1), and on the first day (C2) and the third day (C3) after surgery. The blood sample was anti-coagulated with EDTA and centrifuged at 3,000 rpm for 10 min. The supernatants were subsequently collected and stored at −80°C for subsequent evaluation.

Circulating inflammatory factors, including tumor necrosis factor-alpha (TNF-α, 22122232N, Kexing Biotech, Shanghai, China), interleukin 1 beta (IL-1β, 22122235N, Kexing Biotech, Shanghai, China), and interleukin 10 (IL-10, 22122239N, Kexing Biotech, Shanghai, China), were measured with enzyme-linked immunosorbent assay according to the manufacturer’s instruction.

The secondary outcomes of the study included MAP, HR, and BIS of the patients after entering the operating room (T1), at the induction of anesthesia (T2), 5 min after the induction of anesthesia (T3), at the beginning of the surgery (T4), and 5 min after the beginning of the surgery (T5). Besides, the severity of pain was evaluated with the numeric rating scale (NRS) after the surgery. Other outcomes, such as the incidence of hypoxia and hypercapnia, incidence of post-operative nausea and vomiting (PONV), length of surgery (the time from incision to the completion of suture), accumulating dosages of anesthetics, the number of patients with atropine use in each group, and the incidence of other adverse events were also recorded.

### Sample size estimation

According to the results of our pilot study, the incidence of POCD in elderly patients was approximately 33%, and the incidence of POCD in patients receiving butorphanol was 11% on D1 after surgery. The sample size estimation was performed via the Power Analysis and Sample Size (PASS) 15.0 software. Using a one-sided test, the test efficacy value was set to 80%, and the test level was set to 0.025. The sample size for each group was calculated to be 52 patients. Assuming a 10% dropout rate, at least 57 patients were needed for each group.

### Statistical analyses

The Statistical Product Service Solutions (SPSS) 26.0 software was used for the statistical analysis. Continuous variables that conform to normal distribution were presented as mean and standard deviations (SD) and compared with the t-test. Continuous variables that do not conform to normal distribution were presented as medians (M) and interquartile ranges (IQR) and compared with the U test. Categorized variables were presented as numbers and proportions and compared with the Chi-squared test. Non-parametric tests were used for inter-group and intra-group comparisons. The difference was considered to be statistically significant if *p* < 0.05.

## Results

Between May 2022 and December 2022, 265 older patients scheduled for surgery with general anesthesia in our institution were initially screened. After applying the established inclusion and exclusion criteria, 151 patients were excluded from the study. Among these exclusions, 109 patients did not meet the specified inclusion criteria, and an additional 42 patients declined to provide informed consent. Consequently, a final cohort of 114 patients was enrolled for further investigation.

**Figure 1:**
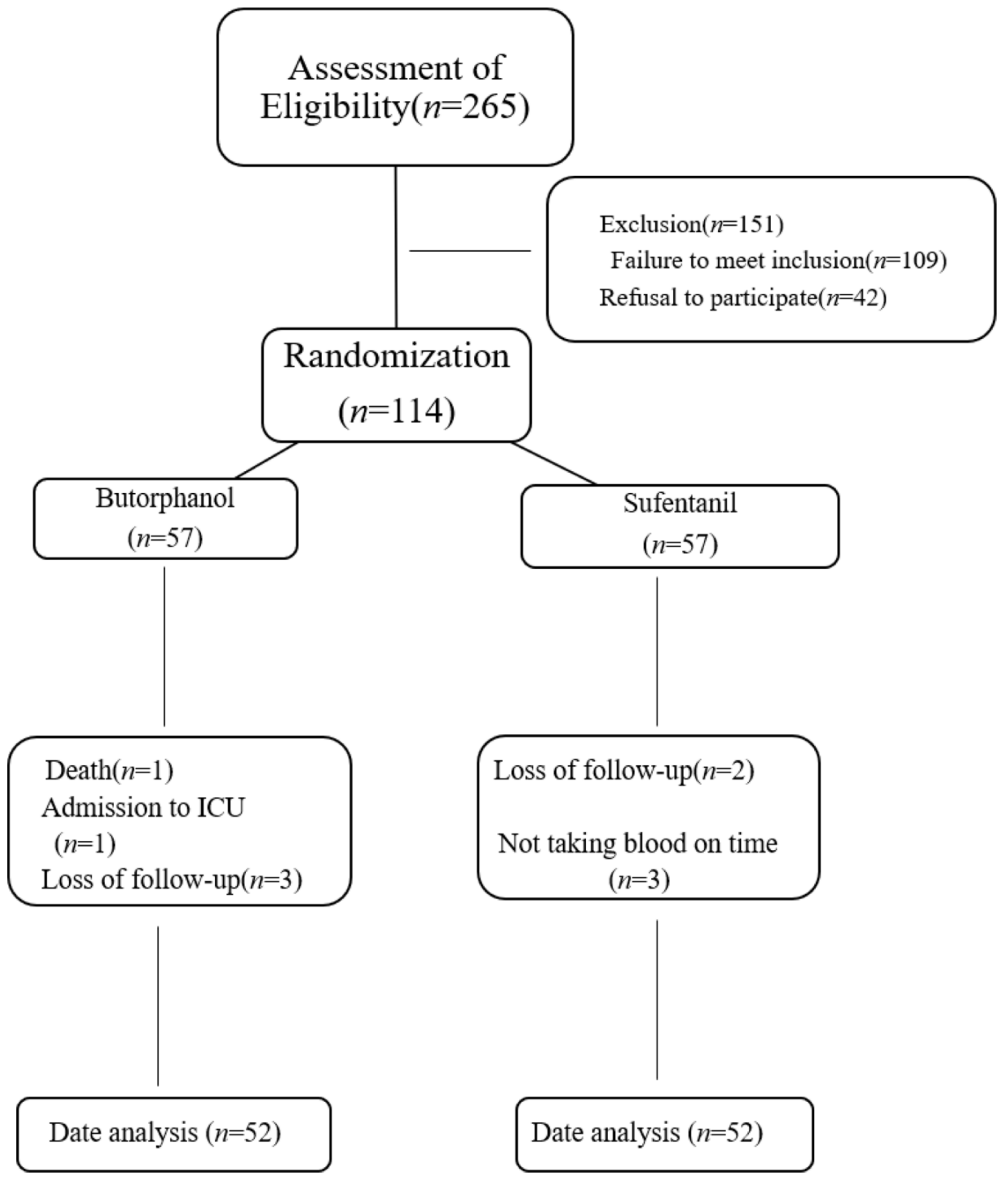
Flowchart of patient inclusion and study procedure.

During the study follow-up, 10 patients were subsequently excluded from the analysis. Among these exclusions, one patient passed away, one patient required admission to the intensive care unit, five patients were lost to follow-up, and three patients did not have their blood samples collected. The final statistical analysis was performed using data from 104 patients, as illustrated in Figure 1.

### Baseline and perioperative characteristics of the included patients

The baseline characteristics of the included patients in each group are shown in Table 1. Generally, 114 patients were included (median age: 71 years, men: 57.7%), and the patients allocated to the butorphanol and the sufentanil groups were well balanced for age, sex, BMI, educational level, ASA class, and comorbidities (*p* all >0.05). These patients received surgeries under general anesthesia, which included general surgeries, orthopedic surgeries, urological surgeries, and gynecological surgeries. The surgical and anesthesia characteristics of the included patients in each group are shown in Table 2. The patients from the two groups were also well matched for surgery type, perioperative characteristics (blood transfusion and length of surgery), anesthetics used etc. (*p* all >0.05).

**Table 1:** Baseline characteristics of the included patients in each group.

	Butorphanol (*n* = 52)	Sufentanil (*n* = 52)	*p* values
Age (years, M [IQR])	71 (67–75)	71 (67–76)	0.956
Men (*n*, %)	28 (53.8)	32 (61.5)	0.427
BMI (kg/m^2^, mean ± SD)	24.13 ± 0.52	22.16 ± 0.45	0.231
Education level (year, M [IQR])	8 (6–9)	7 (6–8)	0.567
ASA Class (*n*, %)			
I–II	29 (55.8)	31 (59.6)	
III	23 (44.2)	21 (40.4)	0.693
Preoperative comorbidities			
Anemia (*n*, %)	18 (34.6)	19 (36.5)	0.838
OSAS (*n*, %)	10 (19.2)	7 (13.5)	0.426
Hypertension (*n*, %)	24 (46.2)	22 (42.3)	0.693
DM (*n*, %)	9 (17.3)	12 (23.1)	0.464
CAD (*n*, %)	2 (3.8)	6 (11.5)	0.270
Arrhythmia (*n*, %)	19 (36.5)	27 (51.9)	0.114
Cerebrovascular disease (*n*, %)	8 (15.4)	3 (5.8)	0.111
Smoking (*n*, %)	9 (17.3)	6 (11.5)	0.402
Alcohol drinking (*n*, %)	3 (5.8)	1 (1.9)	0.610

M, median; IQR, interquartile range; SD, standard deviation; BMI, body mass index; ASA, American Society of Anesthesiologists; OSAS, obstructive sleep apnea syndrome; DM, diabetes mellitus; CAD, coronary artery disease.

**Table 2:** Surgical and anesthesia characteristics of the included patients in each group.

	Butorphanol (*n* = 52)	Sufentanil (*n* = 52)	*p* values
Surgery type (*n*, %)			0.159
General surgery	42 (80.8)	46 (88.5)	
Orthopedic surgery	6 (11.5)	3 (5.8)	
Urological surgery	3 (2.9)	0	
Gynecological surgery	1 (1.9)	3 (2.9)	
Blood transfusion (*n*, %)	2 (3.8)	4 (7.7)	0.674
Length of surgery (min, M [IQR])	165 (131–218)	152 (125–193)	0.252
Hypoxemia (*n*, %)	2 (3.8)	3 (5.8)	1.000
Hypercapnia (*n*, %)	2 (3.8)	2 (3.8)	1.000
Accumulating dose of anesthetics			
Propofol (mg, M [IQR])	450 (338–561)	455 (390–595)	0.303
Cisatracurium besilate (mg, M [IQR])	30 (22–36)	26 (23–33)	0.292
Inhaled sevoflurane (ml, M [IQR])	20 (16–26)	18 (15–23)	0.252
Methoxamine (mg, M [IQR])	6 (3–12)	6 (3–12)	0.789
Patients received atropine (*n*, %)	2 (3.8)	4 (7.7)	0.674

M, median; IQR, interquartile range.

### Primary outcome: early incidence of POCD

**Figure 2:**
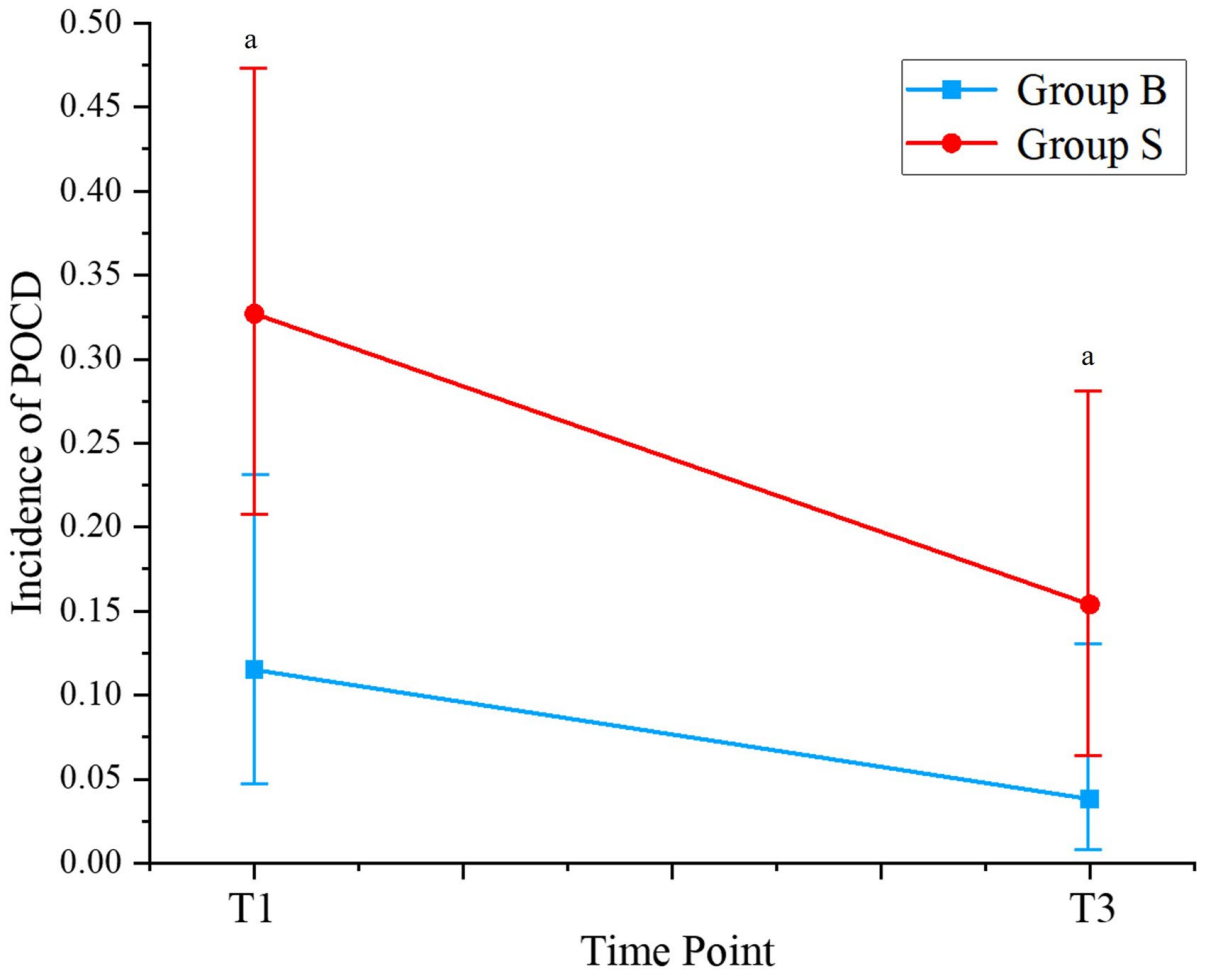
Incidence of POCD at different time points of patients from each group. Group B, patients received butorphanol; Group S, patients received sufentanil; D1, the first day after surgery; D3, the third day after surgery; ^a^*p* < 0.05 compared to patients who received sufentanil; POCD, postoperative cognitive dysfunction.

Changes in perioperative cognitive function, as evaluated by the MMSE and MoCA scores, are shown in Supplementary Table 1. No significant difference in MMSE or MoCA scores was observed between the patients from the two groups on the day before surgery (D0, *p* = 0.96 and 0.47). However, the MMSE and MoCA scores were significantly lower in patients receiving butorphanol than those receiving sufentanil on the first and the third day after surgery (D1 and D3, *p* all <0.05). Consistently, it was found that the incidence of POCD was also lower in patients receiving butorphanol as compared to that of patients receiving sufentanil on the first (D1: 11.5% versus 32.7%, *p* = 0.017) and the third day after surgery (D3: 3.8% versus 15.4%, *p* = 0.046; Table 3 and Figure 2).

**Table 3:** Incidence of POCD at different time points of patients in each group.

Time points	Butorphanol (*n* = 52)	Sufentanil (*n* = 52)	*p* values	Power
D1	6 (11.5)^a^	17 (32.7)	0.017	0.8
D3	2 (3.8)^a^	8 (15.4)	0.046	0.6

Data are presented as numbers and proportions; D1, the first day after surgery; D3, the third day after surgery; ^a^*p* < 0.05 compared to patients of the Sufentanil group; POCD, postoperative cognitive dysfunction.

### Primary outcome: changes in systemic inflammatory cytokines

**Figure 3:**
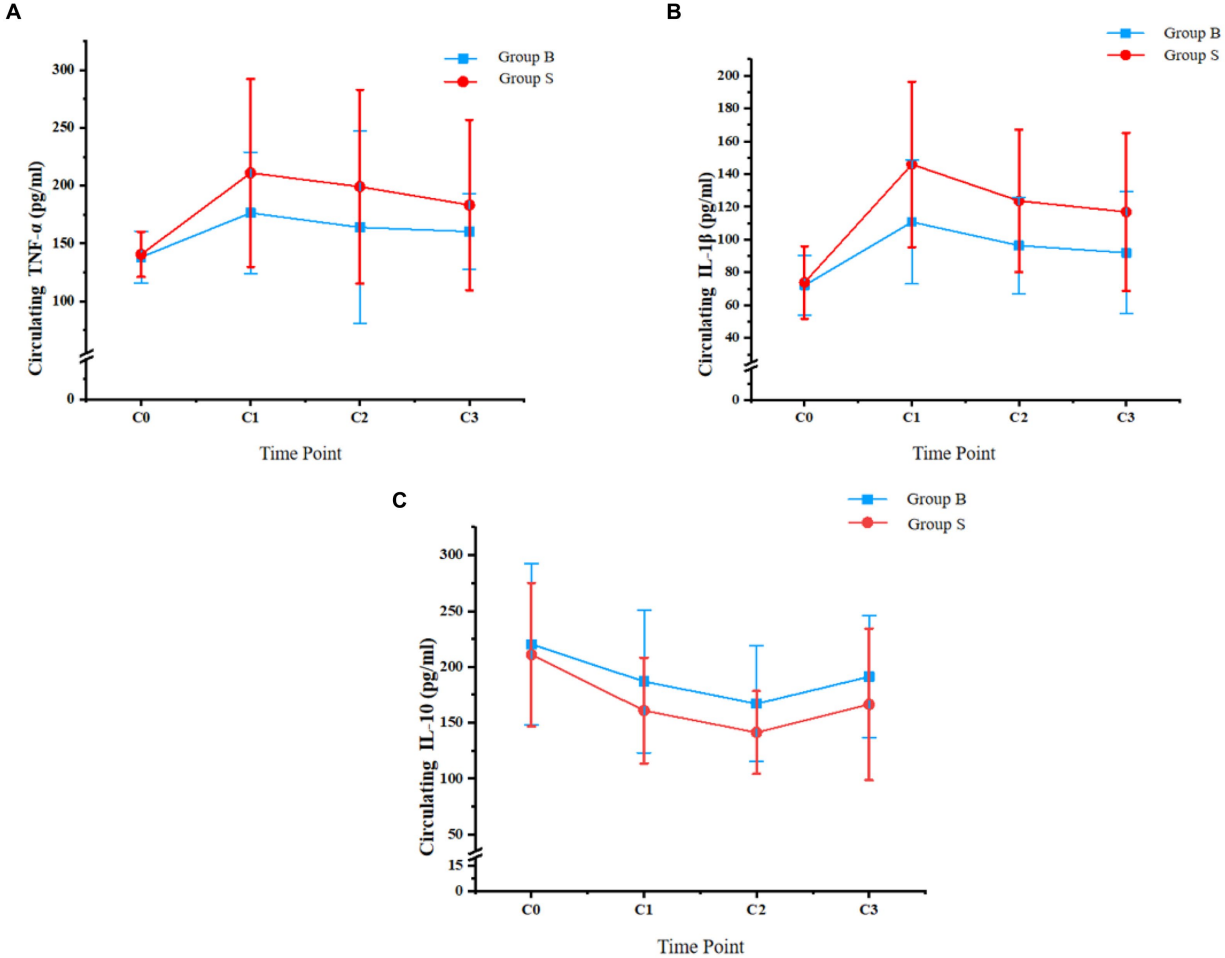
Circulating levels of TNF-α, IL-1β, and IL-10 at different time points of patients from each group; **(A)** circulating TNF-α; **(B)** circulating IL-1β; and **(C)**, circulating IL-10; Group B, patients received butorphanol; Group S, patients received sufentanil; C0, the day before surgery; C1, time of the discharge from post-anesthesia care unit; C2, the first day after surgery; C3, the third day after surgery; TNF-α, tumor necrosis factor alpha; IL-1β, interleukin 1 beta; IL-10, interleukin 10.

Dynamic changes of systematic inflammatory cytokines, including TNF-α, IL-1β, and IL-10, from the day before surgery to the third day after surgery are shown in Figure 3. No significant difference was observed for TNF-α, IL-1β, or IL-10 between the patients from the two groups on the day before surgery (C0, *p* all >0.05). In addition, the circulating levels of TNF-α and IL-1β were significantly lower in patients with butorphanol than those with sufentanil at the discharge from PACU and on the first and third day after surgery (*p* all <0.05). The circulating IL-10 was significantly higher in patients with butorphanol than those with sufentanil at the above three time points (*p* all <0.05).

### Secondary outcomes

The accumulating doses of butorphanol and sufentanil in patients from each group presented as morphine equivalents are shown in Supplementary Table 2, which was comparable between groups (*p* = 0.104).

The changes in hemodynamic parameters, including MAP and HR, from entering OR to 5 min after the beginning of the surgery are shown in Supplementary Figure 1. Compared to the value at T1, the MAP at T2, T3, T4, and T5 of patients in both groups was lower. However, significant differences were observed only at T2, T3, and T4 (*p* < 0.05). Compared to patients receiving sufentanil, patients receiving butorphanol had higher MAP at T1-T5, while the significant between-group difference was only observed at T2, T3, and T4 (*p* < 0.05).

In addition, the HR of patients from both groups declined at T2, T3, T4, and T5 compared to T1 (*p* < 0.05). At T2, the HR of patients receiving butorphanol was significantly higher than that of patients receiving sufentanil (*p* < 0.05), while there were no significant differences in HR between the patients of the two groups at T1, T3, T4, or T5 (*p* > 0.05; Supplementary Figure 1).

Changes in the BIS of patients from each group from entering OR to 5 min after the beginning of the surgery are shown in Supplementary Figure 2. Compared to BIS at T1, the BIS values at T2, T3, T4, and T5 decreased in patients of both groups (*p* < 0.05). At T5, the BIS of patients receiving butorphanol was significantly higher than that of patients receiving sufentanil (*p* < 0.05), while there was no difference in BIS values between patients of the two groups at the other time points (*p* > 0.05).

The incidence of PONV was lower in patients receiving butorphanol compared to patients receiving sufentanil on the first day after surgery (5.8% versus 23.1%, *p* < 0.05; Supplementary Table 3), while no significant difference was observed on the third after surgery (*p* > 0.05). The severity of pain, as evaluated by the NRS, was not significantly different between patients of the two groups on the first or the third day after surgery (*p* > 0.05).

## Discussion

The results of this RCT demonstrate that older surgical patients who received butorphanol exhibited a significantly lower incidence of POCD on both the first and third day after surgery than patients who received sufentanil. Additionally, our investigation revealed that butorphanol administration was associated with decreased circulating levels of TNF-α and IL-1β, along with an increase in IL-10, compared to patients receiving sufentanil. Furthermore, patients administered with butorphanol displayed less affected hemodynamic parameters, such as MAP and HR, and a lower incidence of PONV on the first day after surgery. These collective findings suggest that the use of butorphanol during anesthesia induction could lead to a noteworthy reduction in the early occurrence of POCD in older surgical patients. Notably, this beneficial effect might be attributed to butorphanol’s influence on systemic inflammation, as evidenced by the alterations observed in the levels of inflammatory markers TNF-α, IL-1β, and IL-10.

To the best of our knowledge, this study may be the first clinical trial that compared the influence of butorphanol versus sufentanil on the incidence of POCD in older patients who received surgeries with general anesthesia. Previous studies suggested that many factors may affect the risk of POCD in older patients, such as advanced age, cognitive function before surgery, obesity, length of surgery etc. (Yang et al., 2022). To minimize the potential influences of the related risk factors on the study’s outcome, relatively restrictive inclusion criteria were applied in patient enrollment, which involved limitations of surgical time, BMI, and preoperative MMSE and MoCA scores. Another advantage of the study is that regarding the diagnostic criteria for POCD, the “*Z*-score” definition was used as recommended by the International Study of Post-operative Cognitive Dysfunction (ISPOCD) group (Steinmetz et al., 2010), which may be more universally applied and could be compared between studies.

Currently, the diagnosis of POCD is mainly based on evaluation scales such as MMSE and MoCA. The MMSE evaluates cognitive function with comprehensive aspects, including time, space, immediate memory, delayed memory, calculating ability, attention, and executive ability, which could be achieved within a short duration (5–10 min), making it suitable for evaluating elderly patients (Selvakumar et al., 2022). However, due to the low sensitivity and specificity of MMSE, it is often used with MoCA, which is of high sensitivity and specificity. The MoCA scale can identify patients with mild cognitive impairment, with assessed cognitive functions mainly focused on visual space, naming ability, memory, abstraction, language, and execution, which requires more time for evaluation (Zhuang et al., 2021; Selvakumar et al., 2022). Besides showing that butorphanol was associated with a reduced risk of POCD on the first and third day after surgery. It was also found that the overall cognitive function of patients from both groups recovered gradually in the early phase after surgery. Specifically, patients in our study showed a decrease in MMSE scores on the first and third days after surgery compared to before surgery. The lowest score was observed on the first day after surgery, and the score improved on the third day after surgery. However, it still did not reach the level of the day before surgery. Regarding the MoCA scale scores, there was no upward trend in the scores of the two groups of patients, indicating that the median MoCA scale scores on the first and third post-operative days were similar, both of which were lower than the preoperative MoCA scale scores. These trends were generally consistent with the findings of the previous studies (Qian et al., 2005).

The potential mechanisms underlying the benefits of butorphanol on post-operative cognitive function may be related to its influence on systematic inflammatory factors, based on the findings of our study. An early preclinical study in mice models of orthopedic surgery showed that due to peripheral surgery, the hippocampus experiences an IL-1β-mediated inflammatory response, resulting in memory impairment and the pathogenesis of POCD (Cibelli et al., 2010). Subsequent studies in the same animal model showed that as an upstream regulator of IL-1β, TNF-α provoked the expression of IL-1β in the brain, which may play a key role in mediating the inflammation-related POCD (Terrando et al., 2010). In addition, IL-10, as an anti-inflammatory factor, has been observed to be involved in the protective effect of methane on cognitive impairments in a mouse model of POCD (Zhang et al., 2019). Our results showed that compared to sufentanil, butorphanol significantly increased the circulating level of IL-10 and reduced TNF-α and IL-1β, suggesting anti-inflammation may be an underlying mechanism for the benefits of butorphanol on POCD.

From our perspective, the results that butorphanol exerts a stronger anti-inflammatory effect than sufentanil may be an innovation of this study. Huang et al. (2022) found that butorphanol reduces neuronal inflammatory response by inhibiting p38/JNK/ATF2/p53 signaling. Luan et al. (2021) found that butorphanol may pass through κ Receptors to promote the polarization of macrophages from pro-inflammatory phenotype to anti-inflammatory phenotype, secondary to inhibiting the NF-κ Class B signal pathway. The study on the mechanism of sufentanil pretreatment on lung injury caused by limb ischemia–reperfusion in rats indicates that sufentanil can inhibit NF in lung tissue-κ The expression of B blocks the occurrence of limb ischemia–reperfusion injury, thereby inhibiting the production of inflammatory mediators (Tang et al., 2021). Similarly, some studies have found that sufentanil significantly inhibits expressions of TNF-α and IL-1β (Wu et al., 2022). Future studies with animal or cell experiments may be considered to compare the anti-inflammatory efficacies of butorphanol and sufentanil.

In this study, butorphanol was administered at 40 μg/kg according to previous reports (Parikh et al., 2014), and the accumulating doses of butorphanol and sufentanil between groups presented as morphine equivalents were similar. Compared to sufentanil, butorphanol showed less effect on hemodynamic parameters. This may be because butorphanol predominantly acts on KOR, which is mainly distributed in the cerebral cortex, while sufentanil predominantly acts on μ-opioid receptors (MOR), which are mainly located in the brainstem closely related to respiratory and circulatory functions (Maciejewski, 2012). In addition, it was suggested that the incidence of PONV was lower in patients who received butorphanol than in those with sufentanil. This may be because sufentanil citrate acts on the MOR in the gastrointestinal tract, affecting normal gastrointestinal peristalsis and delaying gastric emptying, which may be associated with a higher risk of PONV (Qian et al., 2022).

In this study, a monitor was connected for close monitoring after the patient returned to the ward. Once respiratory depression occurs, targeted treatment and use of opioid antagonists would be given immediately. However, no respiratory depression was found during follow-up. Due to clinical work environment reasons, the lack of recording of post-operative patient blood oxygen saturation is a limitation of this study. The relationship between blood oxygen saturation and post-operative cognitive function could be studied in the future. Sufentanil is a highly selective μ receptor agonist with a binding force ratio to μ1 receptors compared to μ2 receptors. The effects of μ1 and μ2 receptors are different, with μ1 mainly mediating the analgesic effects and μ2 mainly causing adverse reactions such as nausea, vomiting, and respiratory depression. In addition, respiratory suppression usually occurs when patients are given a large initial dose or when combined with other respiratory-inhibiting drugs. In this study, the sufentanil dosage in the analgesic pump was relatively safe. Moreover, the formula setting for discontinuing the pain pump in this study is also supported by previous studies (Lyu et al., 2020).

This study demonstrates that administering of butorphanol during anesthesia induction, compared to sufentanil, notably reduces the early incidence of POCD and modulates systemic inflammation. This study sheds light on potential avenues for optimizing perioperative care in geriatric surgery. These results suggest that choosing an appropriate opioid during anesthesia induction may not only impact immediate postoperative outcomes but also mitigate the risk of cognitive decline in the elderly population. Furthermore, the observed effects on inflammatory markers highlight the potential role of opioid selection in modulating the inflammatory response, which could have broader implications for the management of surgical stress and recovery. Thus, this study prompts a reevaluation of current anesthetic protocols in geriatric surgery, advocating for the consideration of butorphanol as a preferred option over sufentanil to enhance postoperative cognitive function and mitigate inflammation. Implementing such insights into clinical practice has the potential to improve surgical outcomes and quality of life for elderly patients undergoing surgery.

This study, despite providing valuable insights, is not without its limitations. Firstly, the evaluation of POCD was restricted to a short duration of 3 days after surgery. Future studies should explore the influence of butorphanol on POCD during the entire hospitalization period to better understand its long-term effects on cognitive function. Secondly, due to the small sample size, it was impossible to establish a dose-response relationship between butorphanol and its impact on POCD. Conducting studies with varying doses of butorphanol would be beneficial to discern any potential dose-dependent effects. In addition, cognitive screening tests such as MMSE and MoCA were used for the diagnosis of POCD in this study. However, these cognitive screening tests are generally used to broadly track the possibility of any impairment, rather than to assess cognitive functions. In this regard, the battery of neuropsychological tests may be more reliable than MMSE and MOCA for assessing the cognitive function. Moreover, although the categories of surgeries were matched between groups, this study did not analyze the distribution of individual surgical procedures within the same surgery category. However, different individual surgical procedures may affect the POCD and systematic inflammation differently. The influences of different surgical procedures on the results should be investigated in the future. Lastly, being a single-center open-label study, the results need validation through future multicenter, double-blind RCTs to enhance the robustness and generalizability of the findings. Based on the above limitations, the study should be considered pilot RCTs; high-quality, large-scale RCTs are still needed to validate the findings.

In conclusion, the outcomes of this pilot study demonstrate that butorphanol, when used for anesthesia induction, significantly reduces the early incidence of POCD in older surgical patients compared to sufentanil. This favorable effect might be attributed to its inhibition of systematic inflammation. While these findings hold promise, it is imperative to validate them through high-quality RCTs in the future. Nonetheless, this study provides initial evidence suggesting that the use of butorphanol during anesthesia induction could offer additional benefits for cognitive function in older surgical patients.

## Data availability statement

The original contributions presented in the study are included in the article/Supplementary material, further inquiries can be directed to the corresponding authors.

## Ethics statement

The studies involving humans were approved by the affiliated Hospital of Dalian Medical University. The studies were conducted in accordance with the local legislation and institutional requirements. The participants provided their written informed consent to participate in this study.

## Author contributions

QW: Conceptualization, Data curation, Formal analysis, Visualization, Writing – original draft, Writing – review & editing. DS: Conceptualization, Data curation, Formal analysis, Visualization, Writing – original draft, Writing – review & editing. LY: Conceptualization, Data curation, Formal analysis, Visualization, Writing – original draft, Writing – review & editing. YL: Conceptualization, Data curation, Formal analysis, Visualization, Writing – original draft, Writing – review & editing.

## Footnotes

^1^https://www.chictr.org.cn/showproj.html?proj=184133
