# Supplementary material for: Impact of butorphanol versus sufentanil on postoperative cognition and inflammation in elderly: a pilot study
Source: Front Aging Neurosci. 2024 Jun 7;16:1395725. doi: 10.3389/fnagi.2024.1395725 (PMC11190082; doi:10.3389/fnagi.2024.1395725)
Supplement: Supplementary file 1 [file Data_Sheet_1.docx]

Supplementary Table 1. Changes of MMSE and MoCA Scores at different time points of patients in each group

|  | Time points | Butorphanol  (*n=*52) | Sufentanil  (*n=*52) | *P* values |
| --- | --- | --- | --- | --- |
| MMSE | D0 | 27 (24 - 28) | 27 (24 - 28) | 0.960 |
|  | D1 | 25 (23 - 27) ^ab^ | 24 (22 - 26) ^b^ | 0.022 |
|  | D3 | 26 (24 - 28) ^ab^ | 25 (22 - 26) ^b^ | 0.013 |
| MoCA | D0 | 27 (27 - 28) | 27 (27 - 28) | 0.469 |
|  | D1 | 26 (26 - 26) ^ab^ | 26 (21 - 26) ^b^ | 0.015 |
|  | D3 | 26 (26 - 27) ^ab^ | 26 (26 - 26) ^b^ | 0.007 |

Data are presented as medians and interquartile ranges and compared between and within the groups using the non-parametric test. D0, the day before surgery; D1, the first day after surgery; D3, the third day after surgery; MMSE, the Mini-Mental State Examination; MoCA, the Montreal Cognitive Assessment; a, *P<*0.05 compared to patients of the Sufentanil group; b, *P<*0.05 compared to the score on D0 of patients from the same group

Supplementary Table 2. Accumulating doses of butorphanol and sufentanil in each group presented as morphine equivalents

|  | Butorphanol  (*n=*52) | Sufentanil  (*n=*52) | *P* values |
| --- | --- | --- | --- |
| morphine equivalents (mg, mean*±*SD) | 18.25*±*0.47 | 26.42*±*0.67 | 0.104 |

SD, standard deviation;

Supplementary Table 3. Incidence of PONV and the severity of pain indicated by NRS of patients in each group

|  | Time points | PONV  (*n*, %) | NRS (*n*, %) | |
| --- | --- | --- | --- | --- |
|  |  |  | 1 - 3 | 4 - 6 |
| Butorphanol  (*n=*52) | D1 | 3 (5.8) | 48 (92.3) | 2 (3.8) |
|  | D3 | 0 (0) | 47 (90.4) | 0 (0) |
| Sufentanil  (*n=*52) | D1 | 12 (23.1) | 49 (94.2) | 3 (5.8) |
|  | D3 | 4 (7.7) | 47 (90.4) | 1 (1.9) |

Data are presented as numbers and proportions; D1, the first day after surgery; D3, the third day after surgery; PONV, post-operative nausea and vomiting; NRS, numeric rating scale;

Supplementary Table 4 Results of inflammatory cytokines with effect size and 95% CI

|  |  | Butorphanol  (*n=*52) | Sufentanil  (*n=*52) | P values | Effect sizes | 95%CI |
| --- | --- | --- | --- | --- | --- | --- |
| TNF-α (pg/ml) | C0 | 138.17±22.49 | 140.54±19.60 | 0.568 | -2.371 | -10.577,5.834 |
|  | C1 | 176.47±52.48ab | 211.10±81.39b | 0.011 | -34.618 | -61.256,-7.979 |
|  | C2 | 163.91±83.81ab | 199.16±83.80b | 0.006 | -35.247 | -60.274,-10.220 |
|  | C3 | 160.21±32.87ab | 183.26±73.70b | 0.042 | -23.048 | -45.244,-0.853 |
| IL-1β (pg/ml) | C0 | 72.21±18.02 | 73.95±22.10 | 0.661 | -1.740 | -9.584,6.105 |
|  | C1 | 110.78±37.82ab | 145.90±50.44b | 0 | 35.128 | 17.787,52.468 |
|  | C2 | 96.45±29.32ab | 123.65±43.46b | 0 | 27.205 | 12.786,41.624 |
|  | C3 | 92.02±37.15ab | 116.97±48.18b | 0.004 | 24.955 | 8.219,41.690 |
| IL-10 (pg/ml) | C0 | 220.24±71.90 | 210.93±64.26 | 0.488 | 9.307 | -17.217,35.381 |
|  | C1 | 186.96±63.73ab | 160.93±47.42b | 0.02 | 26.032 | 4.183,47.881 |
|  | C2 | 167.05±51.79ab | 141.31±36.96b | 0.004 | 25.740 | 8.240,43.240 |
|  | C3 | 191.26±54.89ab | 166.48±67.89b | 0.043 | 24.780 | 0.766,48.794 |

a, p < 0.05 between groups; b, p < 0.05 compared to C0 within same group;

CI, confidence interval; TNF-α, tumor necrosis factor alpha; IL-1β, interleukin 1 beta; IL-10, interleukin 10; C0, the day before surgery; C1, time of the discharge from post-anesthesia care unit; C2, the first day after surgery; C3, the third day after surgery;

Supplementary Table 5 Results of MAP with effect size and 95% CI

|  |  | T1 | T2 | T3 | T4 | T5 |
| --- | --- | --- | --- | --- | --- | --- |
| MAP (mmHg) | Butorphanol | 105.19±13.61 | 98.19±21.77^b^ | 91.37±16.95^b^ | 95.15±20.64^b^ | 99.02±15.85 |
|  | Sufentanil | 100.33±11.95 | 85.04±10.49^ab^ | 82.42±10.88^ab^ | 85.19±11.80^ab^ | 94.63±14.17 |
|  | *P* value | 0.056 | 0 | 0.002 | 0.003 | 0.14 |
|  | Effect Size | 4.87 | 13.15 | 8.94 | 9.96 | 4.38 |
|  | 95%CI | -0.12,9.85 | 6.51,19.80 | 3.40，14.48 | 3.42,16.50 | -1.46,10.23 |

a, p < 0.05 between groups; b, p < 0.05 compared to T1 within same group;

MAP, mean arterial pressure; CI, confidence interval; T1, patients in OR before surgery; T2, at the induction of anesthesia; T3, 5 min after the induction of anesthesia; T4, at the beginning of the surgery; T5, 5 min after the beginning of the surgery.

Supplementary Table 6 Results of HR with effect size and 95% CI

|  |  | T1 | T2 | T3 | T4 | T5 |
| --- | --- | --- | --- | --- | --- | --- |
| HR (bpm) | Butorphanol | 74.42±14.85 | 69.83±15.26^ab^ | 65.31±15.74^b^ | 58.13±11.52^b^ | 60.31±12.10^b^ |
|  | Sufentanil | 71.79±12.52 | 63.23±10.61^b^ | 61.00±11.72^b^ | 56.44±10.57^b^ | 59.87±9.87^b^ |
|  | *P* value | 0.33 | 0.012 | 0.117 | 0.437 | 0.839 |
|  | Effect Size | 2.69 | 6.6 | 4.31 | 1.69 | 0.44 |
|  | 95%CI | -2.708,7.977 | 1.484,11.708 | -1.090,9.705 | -2.609,5.993 | -3.853,4.738 |

a, p < 0.05 between groups; b, p < 0.05 compared to T1 within same group;

HR, heart rate; CI, confidence interval; T1, patients in OR before surgery; T2, at the induction of anesthesia; T3, 5 min after the induction of anesthesia; T4, at the beginning of the surgery; T5, 5 min after the beginning of the surgery.

Supplementary Table 7 Results of BIS with effect size and 95% CI

|  |  | T1 | T2 | T3 | T4 | T5 |
| --- | --- | --- | --- | --- | --- | --- |
| BIS | Butorphanol | 94.71±2.69 | 54.90±7.11^b^ | 51.10±5.91^b^ | 50.83±6.39^b^ | 50.46±3.91^ab^ |
|  | Sufentanil | 94.04±2.10 | 56.38±8.46^b^ | 50.35±9.21^b^ | 49.02±7.81^b^ | 45.17±6.87^b^ |
|  | *P* value | 0.158 | 0.337 | 0.622 | 0.199 | 0 |
|  | Effect Size | -0.67 | 1.48 | -0.75 | -1.81 | -5.29 |
|  | 95%CI | -1.611,0.265 | -1.561,4.523 | -3.760.2.260 | -4.583,0.967 | -7.463,-3.114 |

a, p < 0.05 between groups; b, p < 0.05 compared to T1 within same group;

BIS, bispectral index; CI, confidence interval; T1, patients in OR before surgery; T2, at the induction of anesthesia; T3, 5 min after the induction of anesthesia; T4, at the beginning of the surgery; T5, 5 min after the beginning of the surgery.


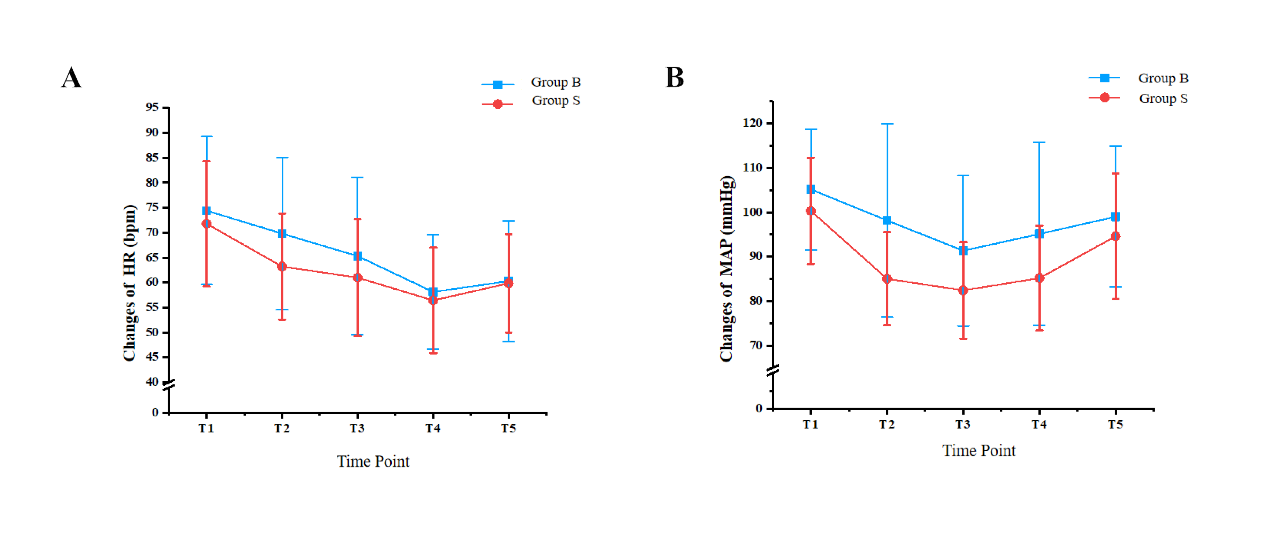


**Supplementary Figure 1. Changes of HR and MAP of patients from each group**; A, changes of HR; and B, changes of MAP

Group B, patients received butorphanol; Group S, patients received sufentanil; T1, patients in OR before surgery; T2, at the induction of anesthesia; T3, 5 min after the induction of anesthesia; T4, at the beginning of the surgery; T5, 5 min after the beginning of the surgery. HR, heart rate; MAP, mean arterial pressure;


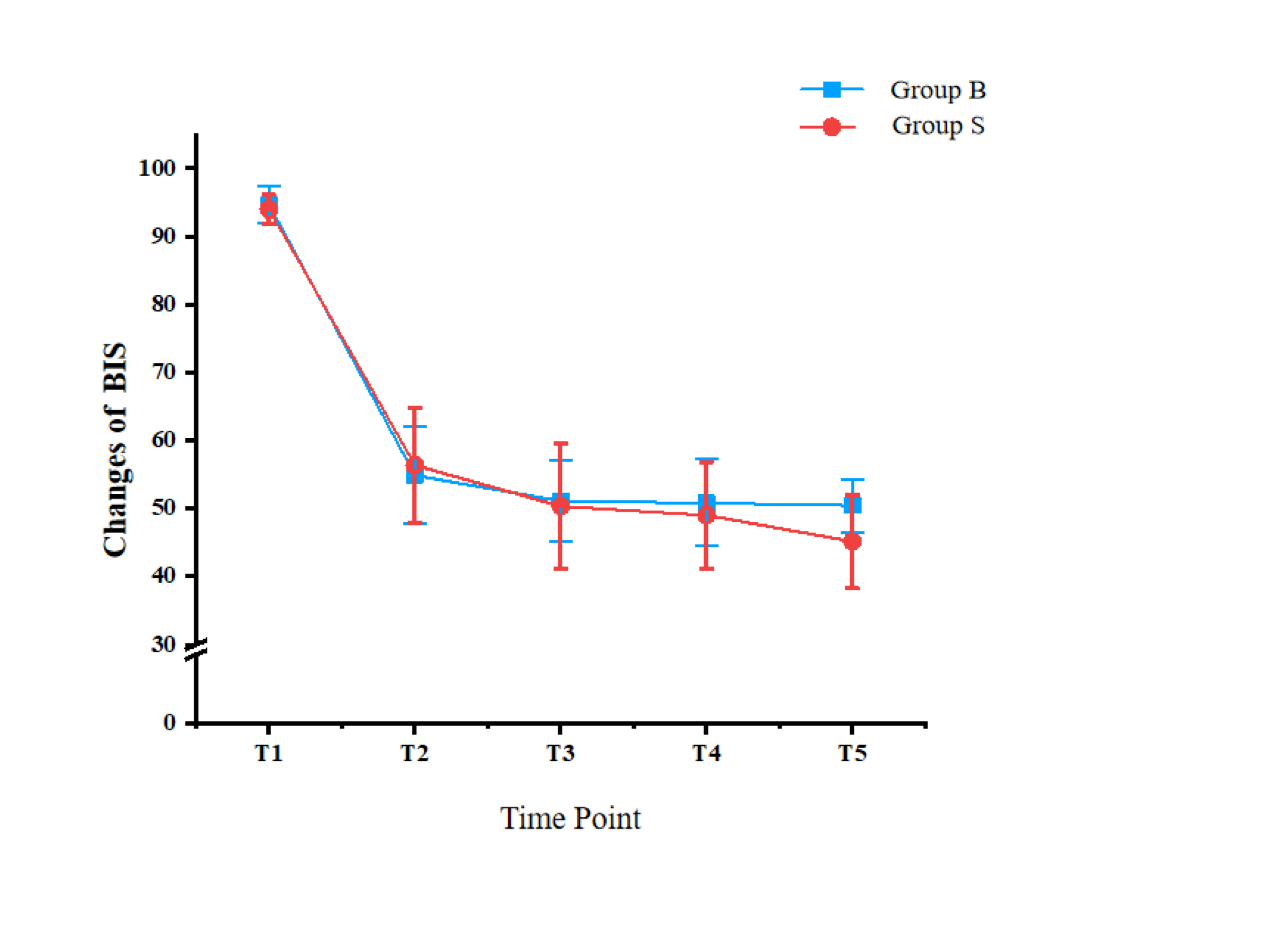


**Supplementary Figure 2. Changes of BIS of patients from each group**

Group B, patients received butorphanol; Group S, patients received sufentanil; T1, patients in OR before surgery; T2, at the induction of anesthesia; T3, 5 min after the induction of anesthesia; T4, at the beginning of the surgery; T5, 5 min after the beginning of the surgery.
